# PGC1α activation by pterostilbene ameliorates acute doxorubicin cardiotoxicity by reducing oxidative stress via enhancing AMPK and SIRT1 cascades

**DOI:** 10.18632/aging.102418

**Published:** 2019-11-16

**Authors:** Dong Liu, Zhiqiang Ma, Liqun Xu, Xiaoyan Zhang, Shubin Qiao, Jiansong Yuan

**Affiliations:** 1 State Key Laboratory of Cardiovascular Disease, Fuwai Hospital, National Center for Cardiovascular Diseases, Chinese Academy of Medical Sciences and Peking Union Medical College, Beijing 100037, China; 2 Department of Thoracic Surgery, Tangdu Hospital, The Fourth Military Medical University, Xi’an 710038, China

**Keywords:** pterostilbene, doxorubicin, acute cardiotoxicity, PGC1α, AMPK, SIRT1

## Abstract

Doxorubicin (DOX) is a widely used and potent anticancer agent, but DOX dose-dependently induced cardiotoxicity greatly limits its use in clinic. Pterostilbene, a natural analog of resveratrol, is a known antioxidant and exerts myocardial protection. The present study explored the action and detailed mechanism of pterostilbene on DOX-treated cardiomyocytes. We investigated the effects of pterostilbene on established acute DOX-induced cardiotoxicity models in both H9c2 cells treated with 1 μM DOX and C57BL/6 mice with DOX (20 mg/kg cumulative dose) exposure. Pterostilbene markedly alleviated the DOX exposure-induced acute myocardial injury. Both *in vitro* and *in vivo* studies revealed that pterostilbene inhibited the acute DOX exposure-caused oxidative stress and mitochondrial morphological disorder via the PGC1α upregulation through activating AMPK and via PGC1α deacetylation through enhancing SIRT1. However, these effects were partially reversed by knockdown of AMPK or SIRT1 *in vitro* and treatment of Compound C (AMPK inhibitor) or EX527 (SIRT1 inhibitor) *in vivo*. Our results indicate that pterostilbene protects cardiomyocytes from acute DOX exposure-induced oxidative stress and mitochondrial damage via PGC1α upregulation and deacetylation through activating AMPK and SIRT1 cascades.

## INTRODUCTION

Doxorubicin (DOX), a member of the anthracyclines, provided the oncologist with highly effective therapeutic regimen to treat tumors, but the unanticipated side-effects associated with acute or chronic cardiotoxicity greatly limits its clinical use [1]. To maximize the overall survival and minimize the cardiac side-effects, DOX-cardiotoxicity has drawn much attention of both cardiologists and oncologists [2]. The primary mechanism of DOX-induced cardiomyopathy is related to the oxidative injury and mitochondrial dysfunction dose-dependent [1, 3]. The cardiomyocyte is especially susceptible to oxidative damage due to the relative lack of biochemical reserves to mitigate oxidation [2, 4]. Therefore, targeting oxidative stress may be a good therapeutic regimen for preventing and treating DOX-cardiotoxicity.

Pterostilbene (3,5-dimethoxy-4′-hydroxystilbene, PTS) is a natural analog of resveratrol and a known antioxidant mainly exists in blueberries and grapes [5, 6]. A dozen of basic studies reveal that pterostilbene is a potent myocardial protective agent against various cardiac diseases, including hypertrophy, diabetic cardiomyopathy, myocardial infarction and ischemia reperfusion injury [7–10]. These favorable effects of pterostilbene application are largely attributed to the free radical scavenging and anti-inflammatory actions [11, 12]. Interestingly, clinical studies indicate that pterostilbene is generally safe for human use and is good for reducing adult blood pressure [13, 14]. However, whether pterostilbene could protect myocardium against DOX-induced acute cardiotoxicity still remains unknown.

Peroxisome proliferator-activated receptor coactivator 1α (PGC1α) is a vital transcriptional coactivator in regulating mitochondrial functions and maintaining mitochondrial homeostasis through mediating the expression of uncoupling protein 2 (UCP2), nuclear respiratory factor 1 (NRF1), etc [4, 15]. The level and activity of PGC1α are particularly vulnerable to DOX exposure thus leading to the DOX-induced mitochondrial damage [15]. The activity of PGC1α could be posttranslational hindered via acetylation [16]. Moreover, PGC1α’s expression and activity are stimulated by its upstream regulator adenosine monophosphate activated protein kinase (AMPK) and sirtuin1 (SIRT1) [2, 4, 17]. Interestingly, AMPK and SIRT1 are also inhibited after DOX exposure in cardiomyocytes [4, 16, 18]. In present study, we aimed to verify whether pterostilbene could exert cardiac protection against acute DOX-cardiotoxicity via the mechanism of alleviating mitochondrial oxidative stress through activating the AMPK/SIRT1-PGC1α cascades.

## RESULTS

### Pterostilbene application alleviated DOX-induced H9c2 cell viability inhibition, mitochondrial damage and oxidative stress

**Figure 1:**
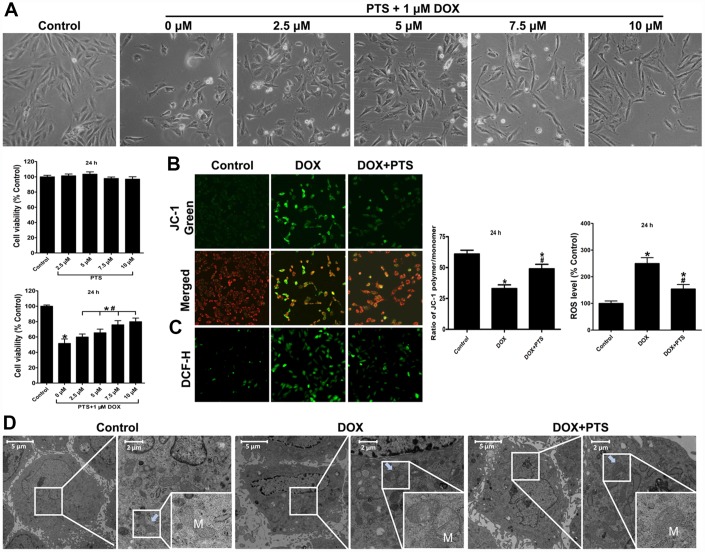
**Effect of pterostilbene treatment on cell viability, mitochondrial membrane potential, ROS generation, mitochondrial morphologic changes in DOX-treated H9c2 cells (24 h).** (**A**) single pterostilbene (2.5-10 μM) treatment and cotreatment of 1 μM DOX with increasing concentrations of pterostilbene (2.5-10 μM) on H9c2 cell viability (24 h). (**B**) mitochondrial membrane potential (ΔΨm) was expressed as the ratio of JC-1 polymer/monomer; red fluorescence represents the mitochondrial JC-1 polymer, and green fluorescence represents the monomeric form of JC-1, indicating ΔΨm depolarization. (**C**) Representative images and ROS level, and the indexes in the control group are defined as 100%. (**D**) Representative images of the ultrastructural morphology of mitochondria in each group of H9c2 cells are shown. The results are expressed as the mean ± SEM. *P < 0.05 vs. the control group, ^#^P < 0.05 vs. the 1 μM DOX-treated group.

**Figure 2:**
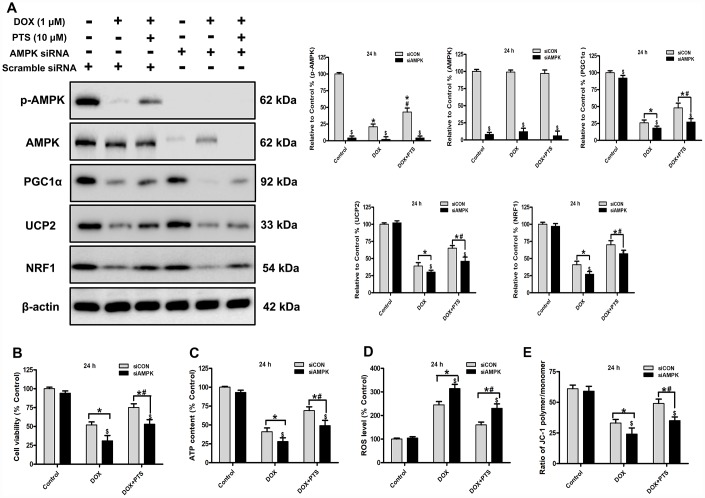
**Effect of pterostilbene treatment combined with AMPK siRNA on cell viability, ATP content, ROS generation and ΔΨm, and AMPK and PGC1α signaling in DOX-treated H9c2 cells (24 h).** (**A**) Representative western blot results of p-AMPK, AMPK, PGC1α, UCP2 and NRF1 are shown. Membranes were re-probed for β-actin expression to show that similar amounts of protein were loaded in each lane. (**B**) Cell viability, (**C**) cellular ATP content, (**D**) ROS level and (**E**) ΔΨm are shown, and (**B**–**D**) three indexes in the Control group of siCON are defined as 100%. The results are expressed as the mean ± SEM. *P < 0.05 vs. the Control group, ^#^P < 0.05 vs. the 1 μM DOX-treated group, ^$^P < 0.05 vs. the siCON group with the representative same drug treatments.

As our previous basic research data suggested [4], the dosage of 1 μM DOX was chosen to carry on the present *in vitro* study. To investigate whether pterostilbene exerts the cardioprotective action against toxicity induced by DOX on H9c2 cells, CCK-8 cell viability assay was conducted to analyze its protective effect. As shown in Figure 1A, pterostilbene treatment (2.5, 5, 7.5 or 10 μM culturing for 24 h) had no effect on H9c2 cell viability, but 1 μM DOX treatment for 24 h significantly inhibited the cell viability. Interestingly, cotreatment of 2.5, 5, 7.5 and 10 μM pterostilbene for 24 h markedly reversed the 1 μM DOX caused decrease of cell viability in a dose-dependent manner. Therefore, this result indicated that pterostilbene exerted cardiac protection against DOX-cardiotoxicity, and 10 μM pterostilbene was chosen to be used for further experimental studies. Generation of ROS thus promoting mitochondrial oxidative stress plays vital actions on the development of cardiac dysfunction. We found 1 μM DOX-exposure for 24 h caused markedly upregulation of ROS level, loss of mitochondrial membrane potential (ΔΨm), and downregulation of ATP content, but pterostilbene cotreatment obviously reversed these DOX-induced mitochondrial oxidative stress by decreasing ROS level and preserving ΔΨm and ATP content (Figure 1B, 1C and Figure 2C). Moreover, the transmission electron microscopic examination on H9c2 cells revealed that 1 μM DOX exposure significantly caused ultrastructural morphology disorder on mitochondria by inducing swelling with cristae disorientation and breakage (Figure 1D). However, pterostilbene cotreatment markedly rescued the myocardial mitochondrion by normalizing the cristae density and architecture (Figure 1D).

### Pterostilbene application activated the AMPK, SIRT1 and PGC1α signaling in DOX-treated H9c2 cells

**Figure 3:**
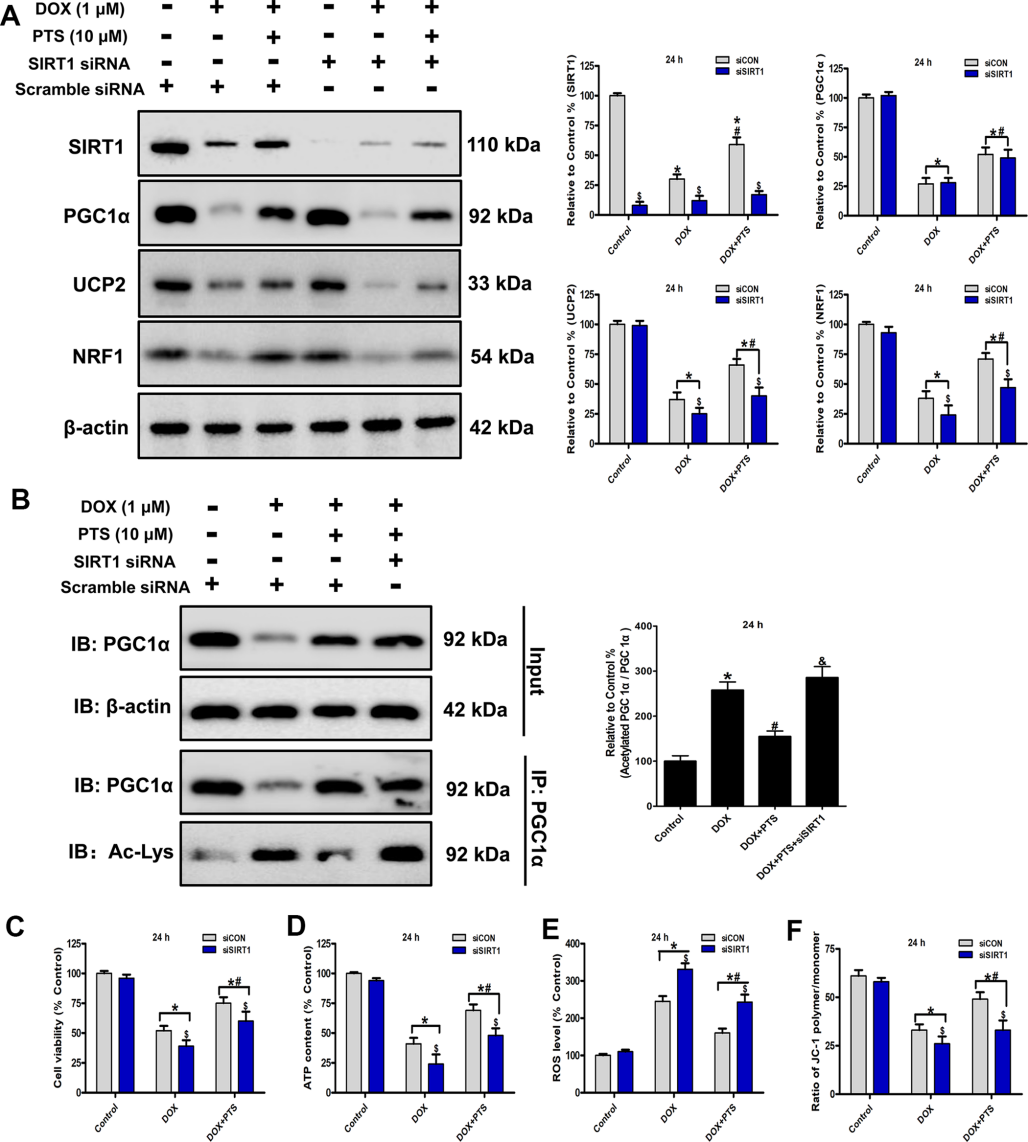
**Effect of pterostilbene treatment combined with SIRT1 siRNA on cell viability, ATP content, ROS generation and ΔΨm, and SIRT1 and PGC1α signaling in DOX-treated H9c2 cells (24 h).** (**A**) and (**B**) Representative western blot results of SIRT1, PGC1α, Ac-PGC1α, UCP2 and NRF1 are shown. Membranes were re-probed for β-actin expression to show that similar amounts of protein were loaded in each lane. IB, immunoblot; IP, immunoprecipitation. (**C**) Cell viability, (**D**) cellular ATP content, (**E**) ROS level and (**F**) ΔΨm are shown, and (**C**–**E**) three indexes in the Control group of siCON are defined as 100%. The results are expressed as the mean ± SEM. *P < 0.05 vs. the Control group, ^#^P < 0.05 vs. the 1 μM DOX-treated group, ^$^P < 0.05 vs. the siCON group with the representative same drug treatments.

To explore the underlying molecular mechanisms regarding myocardial protective actions of pterostilbene on DOX-cardiotoxicity, we further analyzed the expression of p-AMPK, AMPK, SIRT1, and PGC1α and its downstream signaling proteins (NRF1 and UCP2) in H9c2 cells. Consistent with previously reported studies [4], 1 μM DOX-exposure for 24 h significantly inhibited the AMPK activation (AMPK phosphorylation), and decreased the expression of SIRT1, PGC1α, NRF1 and UCP2 (*vs.* Control group, P < 0.05, Figure 2A and Figure 3A). Interestingly, cotreatment of 10 μM pterostilbene markedly reversed the above effects caused by DOX treatment compared with DOX group (P < 0.05, Figure 2A and Figure 3A), suggesting activation of AMPK, SIRT1 and PGC1α signaling cascades may be involved in the actions of pterostilbene-exerted protection against DOX-cytotoxicity.

### Effect of pterostilbene cotreatment with AMPK siRNA on cell viability, oxidative stress parameters, AMPK and PGC1α signaling in DOX-treated H9c2 cells

Our previous study reported that AMPK was a potential upstream regulator on PGC1α signaling, and activation of AMPK-PGC1α cascades was involved in myocardial protection [4]. To investigate the action of AMPK on the myocardial protection of pterostilbene against DOX-cytotoxicity *in vitro,* a specific AMPK siRNA was applicated to knockdown the AMPK expression in the H9c2 cells. Western blot analyses showed that knockdown of AMPK hindered pterostilbene application-exerted increase of PGC1α and its downstream signaling (NRF1 and UCP2) protein levels in H9c2 cells with DOX exposure (*vs.* DOX + PTS group, P < 0.05, Figure 2A). Additionally, compared with the pterostilbene and DOX cotreatment group, AMPK knockdown significantly prevented the pterostilbene cotreatment-exerted rise in H9c2 cell viability, preservation of ΔΨm, cellular ATP content and the decrease of ROS generation with DOX-exposure (P < 0.05, Figure 2B–2E). Therefore, AMPK activation was involved in the pterostilbene-induced heart protection against DOX-cytotoxicity via PGC1α upregulation.

### Effect of pterostilbene cotreatment with SIRT1 siRNA on cell viability, oxidative stress parameters, SIRT1 and PGC1α signaling in DOX-treated H9c2 cells

SIRT1 is a well-known deacetylase and involved in cardiac protection and mitochondrial biogenesis through its posttranslational deacetylation of PGC1α protein [16]. The activity of PGC1α is regulated by the post-translational level, and acetylation of PGC1α leads to the decrease of its transcriptional ability [19]. In present study, we found cotreatment of pterostilbene markedly increased the SIRT1 expression and decreased the acetylated PGC1α level (*vs.* DOX group, P < 0.05, Figure 3A and 3B). To explore the action of SIRT1 on the protective effects of pterostilbene against DOX-induced cardiotoxicity *in vitro,* the SIRT1 was knockdown after SIRT1 specific siRNA application on the H9c2 cells. Western blot analyses revealed that SIRT1 knockdown markedly promoted PGC1α acetylation without influencing PGC1α expression, and downregulated the UCP2 and NRF1 levels in the DOX + PTS + SIRT1 siRNA group compared with the DOX + PTS group (P < 0.05, Figure 3A and 3B). Moreover, compared with the pterostilbene and DOX cotreatment group, SIRT1 knockdown partially reversed the pterostilbene cotreatment-exhibited upregulation of H9c2 cell viability, ΔΨm and cellular ATP content, and the decrease of ROS generation during DOX-exposure (P < 0.05, Figure 3C–3F). These results suggested that SIRT1 participated in the pterostilbene exerted protection against DOX-induced cardiotoxicity via PGC1α deacetylation (activation).

### Effect of cotreatment of pterostilbene with Compound C or EX527 on cardiac toxicity and mitochondrial oxidative stress injury on DOX-impaired mice hearts

**Figure 4:**
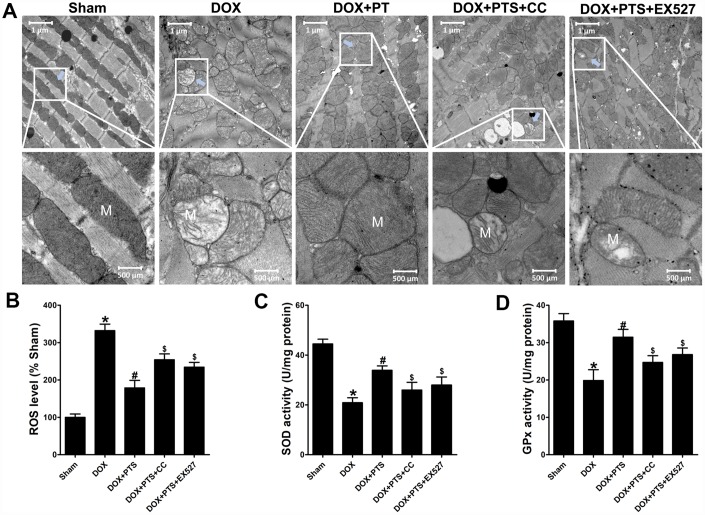
**Effect of pterostilbene treatment combined with Compound C or EX527 on cardiac toxicity and oxidative stress in DOX-exposure mice hearts.** (**A**) Representative images of the ultrastructural morphology of mitochondria from the left ventricular myocardium of experimental mice (magnification: upper panel 9900 ×, lower panel 20,500 ×). (**B**) ROS level, (**C**) SOD and (**D**) GPx activity in the myocardial tissues are showed. The results are expressed as the mean ± SEM. *P < 0.05 vs. the Sham group, ^#^P < 0.05 vs. the DOX-treated group, ^$^P < 0.05 vs. the DOX+PTS cotreated group.

The mitochondrial damage and concomitant oxidative stress injury were considered to be an important cause of DOX-cardiomyopathy. After DOX exposure for 6 days, compared with sham group, transmission electron microscopy examination on heart tissues showed the obvious disorders in ultrastructural mitochondrial morphology including mitochondrial swelling with cristae disorientation and breakage in the DOX-treated hearts (Figure 4A). Interestingly, pterostilbene cotreatment alleviated the DOX-induced injury on mitochondria through normalizing cristae density and architecture (Figure 4A). Moreover, pterostilbene cotreatment markedly downregulated the ROS generation and promoted the SOD and GPx activities in the DOX-exhibited hearts (*vs.* DOX group, P < 0.05, Figure 4B–4D). However, when compared with the DOX + PTS group, cotreatment of compound C (a selective AMPK inhibitor) or EX527 (a selective SIRT1 inhibitor) with pterostilbene clearly mitigated the mitochondrial protective and anti-oxidative effects of pterostilbene on DOX-stimulated hearts (Figure 4A–4D). Pterostilbene application combined with DOX and compound C or EX527 showed severe mitochondrial disorders (Figure 4A), as well as increase of ROS level and decreases in GPx and SOD activities (*vs.* DOX + PTS group, P < 0.05, Figure 4B–4D).

### Effect of cotreatment of pterostilbene with Compound C or EX527 on AMPK, SIRT1 and PGC1α signaling in the myocardial tissue of DOX-stimulated mice

To further confirm the above *in vitro* results that AMPK and SIRT1 activation was involved in the protective effects of pterostilbene against DOX-cytotoxicity via stimulating PGC1α signaling.

**Figure 5:**
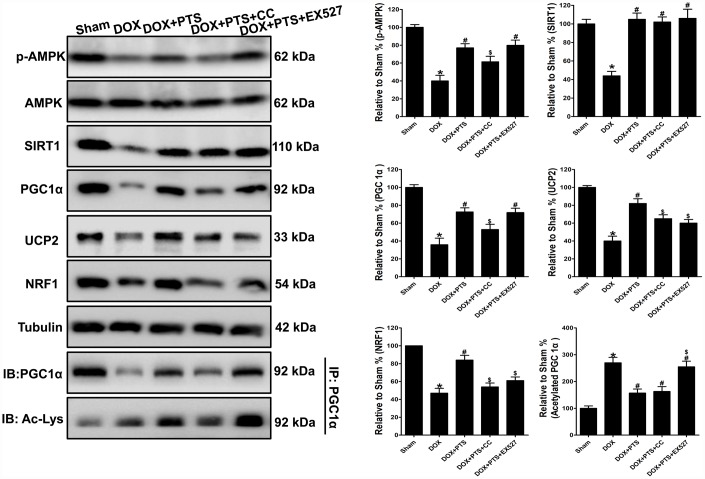
**Effect of pterostilbene treatment combined with Compound C or EX527 on AMPK, SIRT1 and PGC1α signaling protein levels in DOX-stimulated mice hearts.** Representative western blot results of p-AMPK, AMPK, SIRT1, PGC1α, Ac-PGC1α, UCP2 and NRF1 are shown. Membranes were re-probed for Tubulin expression to show that similar amounts of protein were loaded in each lane. IB, immunoblot; IP, immunoprecipitation. The results are expressed as the mean ± SEM. *P < 0.05 vs. the Sham group, ^#^P < 0.05 vs. the DOX-treated group, ^$^P < 0.05 vs. the DOX+PTS cotreated group.

Western blot analyses were applied in the *in* *vivo* study, and we found pterostilbene treatment markedly upregulated the p-AMPK and SIRT1 levels, and inhibited the PGC1α acetylation and promoted the expression of PGC1α and its downstream signaling (NRF1 and UCP2) proteins in DOX + PTS group (*vs.* DOX group, P < 0.05, Figure 5). Interestingly, cotreatment of AMPK inhibitor compound C markedly antagonized pterostilbene induced- upregulation of p-AMPK, PGC1α, NRF1 and UCP2 levels in the DOX + PTS + CC group (*vs.* DOX + PTS group, P < 0.05, Figure 5). Moreover, SIRT1 activity inhibition by EX527 cotreatment significantly reversed pterostilbene promoted PGC1α deacetylation and the increase of NRF1 and UCP2 expression in the DOX + PTS + EX527 group (*vs.* DOX + PTS group, P < 0.05, Figure 5). Taken together, above results indicated that AMPK activation-induced PGC1α upregulation and SIRT1 activation-promoted PGC1α deacetylation were involved in the pterostilbene-exerted myocardial protection against DOX-cytotoxicity.

## DISCUSSION

DOX is a potent anticancer agent and has been widely used in the chemotherapeutic treatment regimens for breast, gastric, thyroid, lung, and ovarian cancers [20]. However, DOX dose-dependently causes acute and chronic cardiotoxicity and this severe side-effect greatly limits its use in clinic for cancer treatment [4, 21]. The molecular mechanism of DOX-cardiotoxicity still remains controversial, but a currently major hypothesis of DOX-induced cardiotoxicity is related to oxidative stress stimulation and mitochondrial injury on cardiac myocyte [4, 22, 23]. Therefore, to mitigate the mitochondrial oxidative damage is one of the therapeutic plans for DOX-induced cardiomyopathy [4, 24, 25]. Resveratrol could exert protection against DOX-induced cardiotoxicity via activating SIRT1 and AMPK signaling cascades thus mitigating oxidative stress, mitochondrial injury, cardiomyocyte apoptosis and cardiac fibrosis induced by DOX exposure [19, 26–29]. Pterostilbene is a natural demethylated analogue of resveratrol and riches in blueberries and grapes [30]. Like resveratrol, pterostilbene functions as a potent antioxidant and exerts myocardial protection [7]. Previous studies reported that pterostilbene could preserve cardiac function against hypertrophy, myocardial infarction and ischemia reperfusion injury via oxidative stress inhibition [7, 8, 10]. Interestingly, our present study found that pterostilbene application significantly reduced DOX-induced injury on H9c2 cells. Moreover, pterostilbene markedly ameliorated doxorubicin exposure-caused acute cardiac tissue damages in mice.

Mitochondria play pivotal roles in maintaining cardiac cells homeostasis and exacerbating injury-induced cell death [31]. Mitochondrial dysfunction disrupts the ATP production, increases the ROS generation, and alters redox balance in cardiomyocytes [4, 32, 33]. Moreover, excess ROS generation also causes irreversible mitochondrial damage and exacerbates cardiac diseases [31]. DOX could accumulate in the inner mitochondrial membrane and thus contributes to mitochondrial toxicity and ROS generation [22]. In present study, pterostilbene cotreatment markedly alleviated DOX-induced mitochondrial swelling with cristae disorientation and breakage both *in vivo* and *in vitro*. Additionally, pterostilbene application also reversed DOX-induced ROS generation and ATP content decrease in H9c2 cells. To sustain cardiac contractile function, cardiomyocytes contain a large volume of mitochondria to preserve ATP generation [31]. Acute DOX exposure for 6 days significantly disrupted mitochondrial function and induced oxidative stress by promoting ROS generation and downregulating the SOD and GPx activities in mice heart tissues. However, treatment of pterostilbene obviously reversed the above adverse effects induced by DOX, suggesting the mitochondrial protective and anti-oxidant actions of pterostilbene on cardiomyocytes.

The transcriptional coactivator PGC1α is critical for maintaining ATP production and mitochondrial function, and regulates a number of mitochondrial genes (e.g., *NRF1* and *UCP2*) [22, 36]. Loss of PGC1α leads to oxidative stress and metabolic dysfunction by disrupting mitochondrial growth and respiration in cardiomyocytes [37, 38]. Under the regulation of PGC1α, NRF1 transcriptionally regulates the expression of mitochondrial function-related genes encoding the respiratory complexes and mitochondrial enzymes [4]. Moreover, UCP2, a downstream factor of PGC1α, is involved in myocardial protection via inhibiting ROS generation [4]. Previous studies found that DOX application markedly inhibited the expression and activity of PGC1α and its downstream factors in cardiac cells [4]. Consistently, our study revealed that acute DOX exposure significantly downregulated the PGC1α, NRF1 and UCP2 levels both in the H9c2 cells and mice heart tissues. However, pterostilbene cotreatment dramatically preserved the expression of PGC1α, NRF1 and UCP2 in DOX-treated H9c2 cells and cardiac tissues. Therefore, activation of PGC1α and its downstream factors may participate in the pterostilbene-exerted protection against DOX-cardiotoxicity.

AMPK is a serine-threonine kinase that functions as a key metabolic regulator for mitochondrial activity and cardiomyocyte energy homeostasis [36, 37]. Activated AMPK by Thr^172^ site phosphorylation exerts myocardial protection against various cardiac diseases, including ischemia reperfusion injury, infarction, and DOX-cardiotoxicity [4, 38, 39]. DOX application could markedly inhibit the AMPK activity in cardiomyocytes [38]. Consistently, our study also revealed that the DOX exposure significantly inhibited AMPK phosphorylation both in H9c2 cells and in mice heart tissues. Pterostilbene has been previously reported as a potent AMPK activator [9], and we verified that treatment of pterostilbene markedly reversed the DOX-induced downregulation of AMPK phosphorylation. PGC1α is one of the downstream molecules of AMPK, and its expression could be regulated by the activity of AMPK [40]. Interestingly, AMPK activation by pterostilbene upregulated the PGC1α expression after DOX exposure both *in vitro* and *in vivo*. However, knockdown of AMPK by siRNA partially impeded the pterostilbene-exerted PGC1α upregulation, cell viability preservation and antioxidant activities in DOX-treated H9c2 cells. Moreover, AMPK inhibitor Compound C treatment also reversed the pterostilbene-exhibited protective actions of mitochondrial morphology protection, antioxidation and PGC1α preservation against acute DOX-cardiotoxicity. These results indicated that activation of AMPK was involved in the myocardial protection of pterostilbene against DOX exposure.

Resveratrol is a classical natural SIRT1 activator and involved in the cardiac protection against DOX-exerted heart injury via SIRT1 activation [28]. Pterostilbene is an analog of resveratrol, but it exhibits better bioavailability and pharmacological activities than resveratrol due to the presence of two methoxy groups which leads to higher cellular uptake and a longer half-life [30]. Moreover, pterostilbene also is a potent natural SIRT1 activator and exerts protection against injuries on various organs through SIRT1 upregulation [41, 42]. SIRT1 functions as a key NAD^+^-dependent deacetylase and increases the activity of PGC1α via posttranslational deacetylation [16, 43, 44]. Our present study found that pterostilbene treatment could reverse the SIRT1 reduction and enhanced PGC1α acetylation induced by DOX-exposure both *in vitro* and *in vivo*. Interestingly, both SIRT1 knockdown *in vitro* and SIRT1 activity inhibition by EX527 *in vivo* markedly hindered the protective activity of pterostilbene and increased the acetylated PGC1α levels in H9c2 cells and mice heart tissues. Thus, above results suggested that the SIRT1-PGC1α cascades activation involved in the protection of pterostilbene against DOX-cardiotoxicity.

## CONCLUSIONS

**Figure 6:**
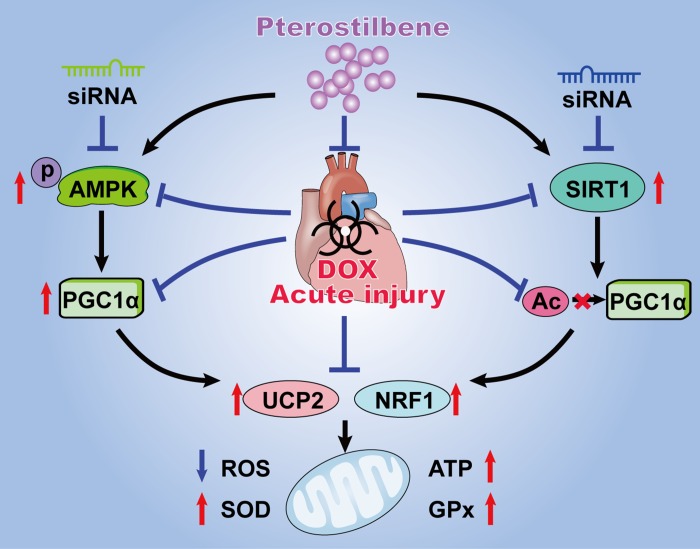
**Schematic diagram summarizing the myocardial protective actions of pterostilbene against acute DOX cardiotoxicity via the PGC1α activation through stimulating AMPK and SIRT1 cascades.** Pterostilbene treatment enhances the AMPK phosphorylation and SIRT1 upregulation thus increasing the PGC1α expression and inhibiting PGC1α acetylation. These effects markedly increase the levels of UCP2 and NRF1, and inhibits oxidative stress via decreasing ROS generation and increasing ATP content, SOD2 and GPx activities in cardiomyocytes.

The present study exhibited the *in vivo* and *in vitro* mechanistic evidence that pterostilbene application could alleviate acute DOX-induced mitochondrial injury and oxidation in cardiomyocytes via promoting AMPK activation and SIRT1 upregulation thus preserving PGC1α activity by upregulated expression and deacetylation (Figure 6). Clinical studies suggest pterostilbene is generally safe at doses up to 250 mg/day for human and is good for cardiovascular system via reducing blood pressure in adults (ClinicalTrials.gov Identifier: NCT01267227) [13, 14]. Moreover, basic researches revealed that pterostilbene could protect heart against hypertrophy, diabetic cardiomyopathy, myocardial infarction and ischemia reperfusion injury [7–10]. To our knowledge, this is the first study reporting the protective actions of pterostilbene against acute DOX-cardiotoxicity. However, further definitive and carefully planned clinical studies are still warranted to verify the cardioprotective effects of pterostilbene against DOX-cardiotoxicity.

## MATERIALS AND METHODS

### Cell culture and siRNA transfection

The cardiomyoblast cell line H9c2 (*Rattus norvegicus*) was purchased from the American Type Culture Collection (ATCC, VA, USA). Cells were cultured in high glucose DMEM medium (Gibco, NY, USA), supplemented with penicillin-streptomycin solution (100 units/ml) (Solarbio, Beijing, China) and 10% fetal bovine serum (FBS, Gibco, USA) according to the providers’ instructions. The commercial rat AMPK siRNA, SIRT1 siRNA and control siRNA were obtained from Santa Cruz Biotechnology (CA, USA). When the H9c2 cells reached 40-60% confluence seeded in the 6-well plates, they were transfected with control or targeted siRNA transfection mixture via using the Lipofectamine 2000 (Invitrogen). Then, cells were cultured in fresh medium after 48 h transfection.

### Cell treatments and in vitro experimental design

Pterostilbene, doxorubicin, and dimethyl sulfoxide (DMSO) were purchased from Sigma-Aldrich (St. Louis, MO, USA). Pterostilbene and doxorubicin stock solutions were maintained in DMSO and diluted in culture medium at certain concentrations for further experiments. The dosage of DOX was chosen based on previously described [4]. The scramble siRNA, AMPK siRNA or SIRT1 siRNA-treated cells were respectively assigned to 3 groups: (i) Control group; (ii) 1 μM DOX treatment group (DOX); (iii) 1μM DOX and 10 μM pterostilbene cotreatment group (DOX + PTS). After those treatments, cells were harvested for further experiments.

### Animals and in vivo experimental design

The animal experiments were performed on healthy adult male C57BL/6 mice at 8 weeks of age; the mice were normally kept in a pathogen-free environment with free access to food and water. All experiments on mice were conducted in accordance with the Guide for the Care and Use of Laboratory Animals published by the U.S. National Institutes of Health (National Institutes of Health Publication No. 85-23, revised 1996). Compound C and EX527 were purchased from Sigma-Aldrich (St. Louis, MO, USA). Pterostilbene, doxorubicin, Compound C and EX527 were dissolved in DMSO and then diluted with normal saline. 75 mice were randomly assigned to five groups (n = 15 for each group): (i) sham-operated control group (Sham); (ii) DOX treatment group (DOX); (iii) DOX and pterostilbene cotreatment group (DOX + PTS); (iv) DOX, pterostilbene and Compound C cotreatment group (DOX + PTS + CC); (v) DOX, pterostilbene and EX527 cotreatment group (DOX + PTS + EX527). DOX was intraperitoneally injected at the dose of 10 mg/kg or the same volume of vehicle (saline) was conducted on day 1 and day 4 for a total of 2 times (20 mg/kg cumulative dose of DOX). Pterostilbene (10 mg/kg/day), Compound C (20 mg/kg/day), EX527 (5 mg/kg/day) or the same volume of vehicle was intraperitoneally injected every day for a total of 7 times (1 day before initial DOX treatment). All mice were euthanized 6 days after the initial injection of DOX. The dosages of DOX, pterostilbene, Compound C and EX527 were chosen based on the previous reports [4, 45, 46].

### Cell viability analysis

After the different treatments on H9c2 cells for 24 h, the cell viability was detected via using the CCK-8 kit (7Sea, Shanghai, China) according to the previously described [47]. Optical density (OD) values were measured at 450 nm through the microplate reader (SpectraMax 190, Molecular Device, USA). Cell viability was presented as the ratio of OD values compared with the Control group, and representative images were taken by a 600D camera (Canon Company, Japan).

### Cell mitochondrial membrane potential analysis

The H9c2 cells’ mitochondrial membrane potential (ΔΨm) was measured by the fluorescent dye JC-1 staining. After different treatments, cells were incubated with JC-1 working solution for 20 min in the dark at 37 °C. Cells exhibiting red fluorescence are in normal ΔΨm state. Green fluorescence represented the monomeric form of JC-1 and suggested ΔΨm depolarization. Images were obtained using the confocal microscope, and the results were analyzed by the SpectraMax 190 microplate reader, and expressed as the proportion of aggregated JC-1 and monomeric JC-1.

### Cellular ATP content analysis

Cellular ATP concentrations were assayed with an ATP detection kit (Beyotime Institute of Biotechnology, China). H9c2 cells were lysed through the ATP assay buffer according to the instructions of the manufacturer. The level of ATP was measured by the emitted light of the mixture of the 50 μl cell lysis supernatant and 50 μl luciferase reagent using the microplate reader.

### ROS, SOD and GPx determination

To analyze the oxidative stress parameters, the ROS level and the GPx and SOD activities in cultured cells and heart tissues were detected via using commercially available DCFH-DA (Beyotime Institute of Biotechnology, China), SOD and GPx assay kits (Nanjing Jiancheng Bioengineering Institute, China) according to the manufacturer’s instructions. The data were analyzed by the SpectraMax 190 microplate reader (Molecular Device, USA).

### Transmission electron microscopy

The ultrastructure of mitochondria of H9c2 cells and mouse heart samples were observed via using the transmission electron microscopy. After different treatments, cells were collected and fixed in 2% glutaraldehyde for 24 h at 4 °C, and then cells were diced into 2 mm cubes. 6 days after the initial injection of DOX, the mouse hearts were cut into 2 mm cubes placed in 2% glutaraldehyde at 4 °C for 24 h. Later, both the cell and tissue samples were cut into 75 nm thickness sections, then they were stained with uranium acetate and lead citrate; images were observed using a TECNAI G2 Spirit Biotwin 120 kV electron microscope.

### Western blot and immunoprecipitation

After various treatments, the cells and heart samples were collected and lysed by the RIPA buffer; a BCA protein assay was used to analyze the protein concentration (Thermo Scientific, Rockford, IL, USA). As for immunoprecipitation, equal amounts of cell or tissue extracts were incubated with 2 ug anti-PGC1α (Santa Cruz, sc-518025) at 4 °C for 12 h on a rotating incubator, and then the samples were further incubated with the 20 μl Protein A/G Agarose (Beyotime Institute of Biotechnology, China) at 4 °C for 2 h rotation. Immunocomplexes were washed four times with NETN buffer before diluted with SDS-PAGE sample loading buffer. Then, the boiled protein samples of each group were fractionated by SDS-PAGE and transferred to PVDF membranes. Later, After blocked with 5% nonfat milk in TBST for 2 h at room temperature, the membranes were incubated with primary antibodies against AMPK (CST, #5831, 1:1000), p-AMPK Thr^172^ (CST, #50081, 1:1000), PGC1α (Abcam, ab54481, 1:1000), Ac-Lys (CST, #9441, 1:1000), NRF1 (Abcam, ab175932, 1:1000), UCP2 (Abcam, ab97931, 1:500), β-actin (Abcam, ab6276, 1:3000) and Tubulin (CST, #2148, 1:3000) for 12 h at 4 °C. Thereafter, the membranes were washed with TBST and exposed to the corresponding secondary antibodies (1:5000, Zhongshan Company, China) at room temperature for 2 h. The fluorescent signal was detected using a BioRad imaging system (BioRad, Hercules, CA, USA), and the signal was quantified using Image Lab Software (BioRad, Hercules, CA, USA).

### Statistical analyses

IBM SPSS 23.0 (SPSS Inc., Chicago, USA) software was used for further data analyses. In this study, between-group comparisons, one-way ANOVA followed by Bonferroni post hoc test. Data are presented as the means ± SEM, and the *P* value < 0.05 was considered as statistically significant.
